# Graphene Oxide@3D Hierarchical SnO_2_ Nanofiber/Nanosheets Nanocomposites for Highly Sensitive and Low-Temperature Formaldehyde Detection

**DOI:** 10.3390/molecules25010035

**Published:** 2019-12-20

**Authors:** Kechuang Wan, Jialin Yang, Ding Wang, Xianying Wang

**Affiliations:** School of Materials Science and Engineering, University of Shanghai for Science & Technology, No. 516 JunGong Road, Shanghai 200093, China; 173762642@st.usst.edu.cn (K.W.); jialin_yang@foxmail.com (J.Y.); xianyingwang@usst.edu.cn (X.W.)

**Keywords:** GO@SnO_2_ NF/NSs, nanocomposites, formaldehyde gas sensors, three-dimensional nanostructure

## Abstract

In this work, we reported a formaldehyde (HCHO) gas sensor with highly sensitive and selective gas-sensing performance at low operating temperature based on graphene oxide (GO)@SnO_2_ nanofiber/nanosheets (NF/NSs) nanocomposites. Hierarchical SnO_2_ NF/NSs coated with GO nanosheets showed enhanced sensing performance for HCHO gas, especially at low operating temperature. A series of characterization methods, including X-ray diffraction (XRD), Field emission scanning electron microscopy (FE-SEM), Transmission electron microscope (TEM), X-ray photoelectron spectroscopy (XPS) and Brunauer–Emmett–Teller (BET) were used to characterize their microstructures, morphologies, compositions, surface areas and so on. The sensing performance of GO@SnO_2_ NF/NSs nanocomposites was optimized by adjusting the loading amount of GO ranging from 0.25% to 1.25%. The results showed the optimum loading amount of 1% GO in GO@SnO_2_ NF/NSs nanocomposites not only exhibited the highest sensitivity value (R_a_/R_g_ = 280 to 100 ppm HCHO gas) but also lowered the optimum operation temperature from 120 °C to 60 °C. The response value was about 4.5 times higher than that of pure hierarchical SnO_2_ NF/NSs (R_a_/R_g_ = 64 to 100 ppm). GO@SnO_2_ NF/NSs nanocomposites showed lower detection limit down to 0.25 ppm HCHO and excellent selectivity against interfering gases (ethanol (C_2_H_5_OH), acetone (CH_3_COCH_3_), methanol (CH_3_OH), ammonia (NH_3_), methylbenzene (C_7_H_8_), benzene (C_6_H_6_) and water (H_2_O)). The enhanced sensing performance for HCHO was mainly ascribed to the high specific surface area, suitable electron transfer channels and the synergistic effect of the SnO_2_ NF/NSs and GO nanosheets network.

## 1. Introduction

HCHO is a colorless gas with pungent smell, which was considered as one of the most serious indoor air pollutants [1]. As a critical raw material, HCHO has been widely applied for various areas of industry, such as construction materials, daily products and so on [2]. Simultaneously, when people are exposed to the certain concentration of HCHO, a series of reactions may be caused due to its high toxicity, such as corrosion of the gastrointestinal tract; inflammation of the mouth, eye or nose; throat irritant reaction and so on. Especially, nose and throat cancer are more easily caused than what is expected [3,4]. In order to detect HCHO effectively, various techniques and metal oxide semiconductors (MOSs) based gas sensors have recently developed, including SnO_2_ [5,6,7], Co_3_O_4_-ZnO core-shell NFs [8], ultrathin In_2_O_3_ nanosheets [9], reduced graphene oxide (rGO)/TiO_2_ [10] and Cr-doped WO_3_ nanosheets [11]. Due to their stable sensitivity and low-cost production, metal oxide semiconductors are widely studied. However, these gas sensors based on MOSs have many disadvantages, including low sensitivity, poor selectivity and/or relatively high optimum operation temperature. Hence, designing and developing gas sensors with high sensibility, excellent selectivity and lower optimum operation temperature is urgent and important.

Graphene is a typical two-dimensional (2D) sheet of sp^2^ bonded carbon with excellent electronic applications. Due to its unique physical and chemical properties, many efforts have been carried out on the application of graphene as sensing elements [12]. These advantages, including its high conductivity, large surface area and low electrical noise, make it a promising platform for preparing new sensors [13,14,15]. In order to prepare a new gas sensor with high sensing performance, low operation temperature and excellent selectivity, the combination of graphene and metal oxide semiconductors is a new strategy to enhance sensing performance compared to pure sensing materials [16]. Gaikwad et al. have reported a NH_3_ gas sensor based on Polyaniline/Graphene Oxide (PANI/GO) by nanoemulsion method [17]. Sun et al. have synthesized rGO/ZnSnO_3_ composites as a sensing material for detecting HCHO gas by a facile solution-based self-assembly synthesis method [18]. Rong et al. have prepared microstructures of SnO_2_@rGO nanocomposites for HCHO detection by facile thermal treatment [5]. Feng et al. have reported excellent ammonia sensors based on rGO/Co_3_O_4_ nanofibers by simple electrospinning [19]. Guo et al. have demonstrated excellent acetone sensors by using the electrospun rGO/Fe_2_O_3_ NFs as a sensing material [20]. However, these sensors based on nanocomposites showed low sensitivity, higher operation temperature and poor selectivity. Naturally, preparing a HCHO gas sensor with excellent sensing performance at low operation temperature is urgent and essential.

In this work, we reported an excellent HCHO gas sensor based on 3D hierarchical SnO_2_ NF/NSs coated by the GO nanosheets. The hierarchical SnO_2_ NF/NSs was prepared by facile electrospinning and further hydrothermal methods, and then, GO was impregnated as the sensitizer. The structure and morphology of nanocomposites were studied by a series of characterizations, including X-ray diffraction (XRD), field emission scanning electron microscopy (FE-SEM), transmission electron microscope (TEM), X-ray photoelectron spectroscopy (XPS) and Brunauer–Emmett–Teller (BET). Gas sensor evaluating system was used to investigate the sensing performance of GO@SnO_2_ NF/NSs sensors, including the response value, the optimum operation temperature, dynamic response/recovery process and selectivity. Furthermore, to confirm the optimal loading ratio of GO, the sensing performance of SnO_2_ NF/NSs with different loading ratios of GO was also investigated. Finally, the sensing mechanism of GO@SnO_2_ NF/NSs and the role of GO were also discussed.

## 2. Results and Discussions

### 2.1. Characterization of Sensing Materials

**Figure 1:**
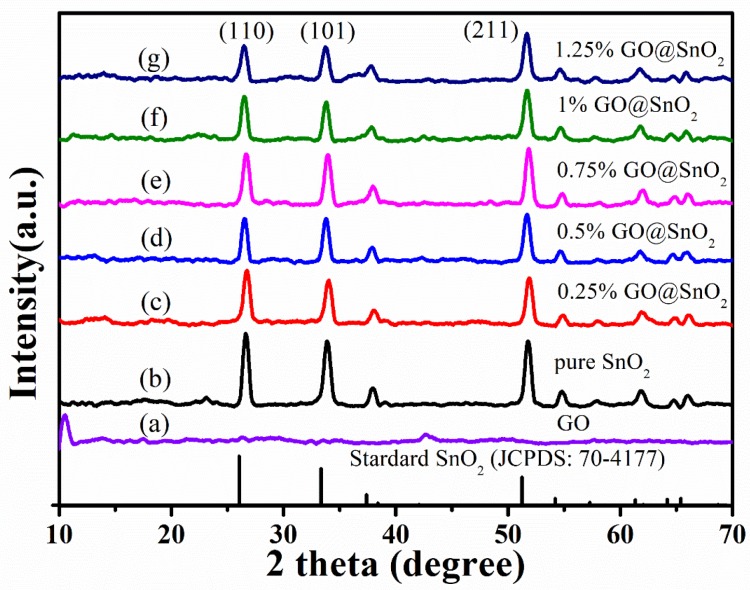
XRD patterns of (**a**) GO, (**b**) SnO_2_ NF/NSs and (**c**–**g**) GO@SnO_2_ NF/NSs nanocomposites with different GO content.

The XRD patterns of pristine SnO_2_ NF/NSs, GO@SnO_2_ NF/NSs nanocomposites and GO were presented in Figure 1. The diffraction peaks of SnO_2_ are not changed after introducing GO nanosheets. The main diffraction peaks observed at 2θ = 26.6°, 33.8°, 37.9° and 51.7° correspond to (110), (101), (200) and (211) peaks of SnO_2_ with a tetragonal rutile structure (PDF#70-4177), respectively. In addition, the GO@SnO_2_ NF/NSs has similar diffraction peaks to pristine SnO_2_. The diffraction peak centered at 2θ = 10.6° in the XRD spectra was signed as (002) of GO [21]. However, the diffraction peak of (002) was not obvious in the XRD spectra of GO@SnO_2_ NF/NSs nanocomposites, which due to the low loading ratio the weak peak intensity of GO [22,23,24]. In addition, the absence of XRD characteristic peaks of GO further indicated their good dispersity in nanocomposites. It is worth noting that the diffraction peak intensities of GO@SnO_2_ NF/NSs nanocomposites gradually weaken compared with pristine SnO_2_, which is attributed to the destroyed orderliness during stacking of GO/SnO_2_ nanocomposite networks [6,21]. No other diffraction peak of impurity was observed in the XRD spectra of all products.

**Figure 2:**
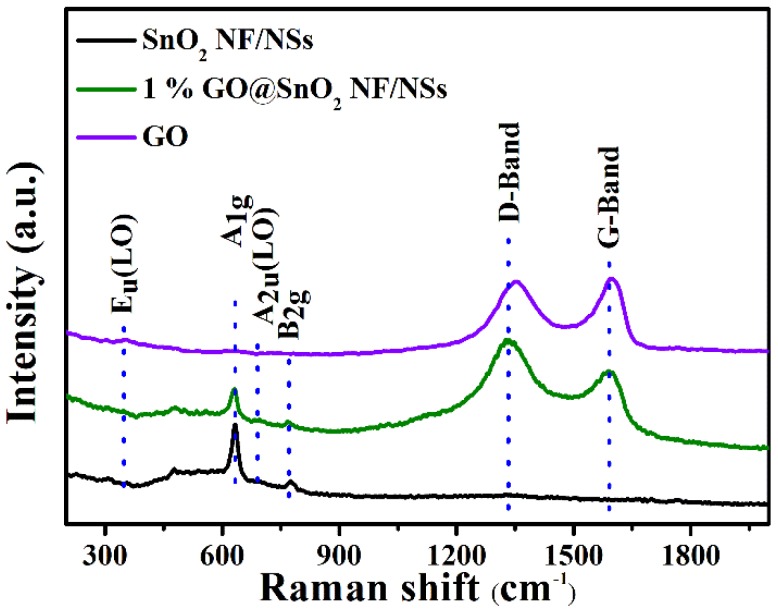
Raman spectra of pure SnO_2_ NF/NSs, 1% GO@SnO_2_ NF/NSs and pure GO nanosheets.

Raman spectra with 633 nm laser was employed to testify the presence of GO in GO@SnO_2_ NF/NSs nanocomposites. Figure 2 showed the Raman spectra of SnO_2_ NF/NSs, 1% GO@SnO_2_ NF/NSs and GO. Compared with pure SnO_2_ NF/NSs, two strong peaks were observed at 1327 and 1590 cm^−1^, which match the D and G bands of GO, respectively. The observed G peak contains information regarding the sp^2^ hybridization within the carbon materials, and the D peak is used to measure disordered degree and the induced defect of GO due to the presence of functional groups [25,26]. The intensity ratio of D band and G band suggests the disorder degree and the average size of the sp^2^ dominating in graphite [27]. This suggests the presence of GO in GO@SnO_2_ NF/NSs nanocomposites. What is more, the ratio of D and G intensity in 1% GO@SnO_2_ NF/NSs is higher than GO, which responds to the reported study [28,29]. The measured result showed that the interaction between GO and SnO_2_ NF/NSs leads to the increase of defects in GO nanosheets, and the process can be acted as a slight reduction from GO to rGO [29,30,31].

**Figure 3:**
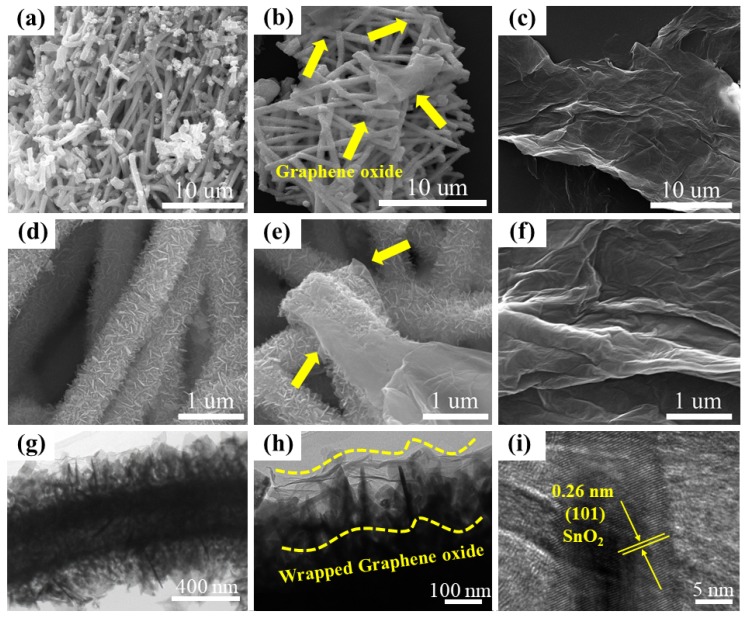
SEM images of the as-prepared (**a**) and (**d**) SnO_2_ NF/NSs, (**b**) and (**e**) 1% GO@SnO_2_ NF/NSs and (**c**) and (**f**) GO; (**g**) low magnification TEM image; (**h**) low magnification TEM image, (**i**) HRTEM image of the 1% GO@SnO_2_ NF/NSs nanocomposites.

SEM was conducted to investigate the morphologies of pristine SnO_2_ NF/NSs, 1% GO@SnO_2_ NF/NSs and GO nanosheets. As observed in Figure 3a,d, the diameter and length of hierarchical SnO_2_ NF/NSs are about 700 nm and tens of micrometers, respectively. Furthermore, SnO_2_ nanosheet arrays vertically grew and were uniformly distributed on the surface of SnO_2_ nanofibers with high length–diameter ratio. The special orientation and open structure expose the whole surface to the gas atmosphere, which can enhance surface active sites and improve the absorption ability to target gas molecules. As can be seen in Figure 3c,f, GO showed gossamery nanosheet structure with slight and disordered wrinkles, which makes it produce a large specific surface area [24]. Figure 3b,e displayed the SEM images of 1% GO@SnO_2_ NF/NSs. Obviously, hierarchical SnO_2_ NF/NSs were coated by GO nanosheets, and a few of GO nanosheets were embedded into interspace among these SnO_2_ NF/NSs. It is noteworthy that GO nanosheets showed a crumpled, rippled morphology and adhered to the surface of pure SnO_2_ NF/NSs tightly. In addition, GO nanosheets evenly distributed in hierarchical SnO_2_ NF/NSs and formed network. Compared with pure hierarchical SnO_2_ NF/NSs, the intersecting bonding and good contacts between SnO_2_ NF/NSs and GO nanosheets can generate more electrical connection paths, which can significantly improve the electron transfer in gas sensing test [21]. Moreover, these special structures constructed by hierarchical SnO_2_ NF/NSs and GO nanosheets can make the diffusion and adsorption of gas molecules more effective than that of the pure SnO_2_ NF/NSs, which can contribute to the improvement of gas-sensing performance [32]. To further study the microstructure of GO@SnO_2_ NF/NSs nanocomposites, TEM and high-resolution TEM (HRTEM) were carried out and shown in Figure 3g–i. Figure 3g showed the TEM image of GO@SnO_2_ NF/NSs nanocomposites, which demonstrated that GO nanosheets had successfully been loaded on the surface of hierarchical SnO_2_ with a diameter of about 700 nm. As can be seen in Figure 3g,h, thin and wrinkled GO nanosheets adhered to the surface of SnO_2_ nanosheet arrays. There is a good contact and intersecting bonding between hierarchical SnO_2_ NF/NSs and GO nanosheets, which corresponds to the observed SEM result of the GO@SnO_2_ NF/NSs from Figure 3b,e. The lattice space of 0.26 nm measured in Figure 3i matches well with the (101) planes of tetragonal rutile SnO_2_. No other impure lattice was observed, which was consistent with the XRD analysis.

**Figure 4:**
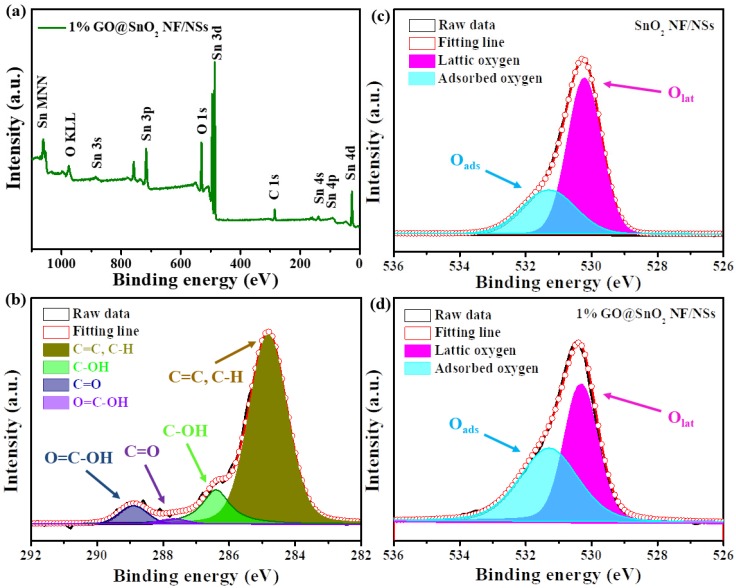
XPS spectra of SnO_2_ NF/NSs and 1% GO@SnO_2_ NF/NSs nanocomposites: (**a**) survey of 1% GO@SnO_2_ NF/NSs nanocomposite, (**b**) C 1s of 1% GO@SnO_2_ NF/NSs nanocomposites, (**c**,**d**) O 1s of pure SnO_2_ NF/NSs and 1% GO@SnO_2_ NF/NSs nanocomposites, respectively.

In order to investigate the chemical composition and oxidation state of the sensitive materials, XPS was conducted. As shown in Figure 4a, the spectrum of GO@SnO_2_ NF/NSs indicated that the main constituent elements were C, Sn and O in the GO@SnO_2_ NF/NSs nanocomposites. Figure S1 showed that the two peaks centered at the binding energy of 486.3 eV and 494.7 eV corresponded to Sn 3d_5/2_ and Sn 3d_3/2_ in pure SnO_2_ NF/NSs, respectively, which implies the existence of Sn^4+^ [33]. The strong peaks located at the binding energy of 486.5 eV and 494.9 eV are attributed to Sn 3d_5/2_ and Sn 3d_3/2_ in GO@SnO_2_ NF/NSs nanocomposites, respectively. Figure 4b shows C 1s spectrum of GO@SnO_2_ NF/NSs with four peaks at 284.8 eV, 286.4 eV, 287.7 eV and 288.9 eV, which are ascribed to the C-C, C-OH, C=O and O=C-OH, respectively [34]. Gas adsorption ability of sensing material is crucial for sensing performance. To further demonstrate the oxygen species of the obtained sensing materials, the O 1s spectrum of sensing materials was analyzed. The O 1s spectrum of pure SnO_2_ NF/NSs and 1% GO@SnO_2_ NF/NSs were shown in Figure 4c,d, respectively. The O 1s XPS spectra of pure SnO_2_ NF/NSs and 1% GO@SnO_2_ NF/NSs could be fitted into two peaks, which correspond to lattice oxygen (O_lat_) and adsorbed oxygen (O_ads_). The latter peaks centered at the binding energy of 530.2 ± 0.1 eV could be ascribed to lattice oxygen, and the former peaks located at the binding energy of 531.3 ± 0 eV belonged to chemisorbed oxygen species (O^−^, O_2_^−^ or O^2−^) on the surface of sensing material. As shown in Table 1, the relative percentage of different oxygen species on the surface of pure SnO_2_ NF/NSs and 1% GO@SnO_2_ NF/NSs have been summarized. The 1% GO@SnO_2_ NF/NSs showed smaller O_lat_ percentage (49.14%) than that of pure SnO_2_ NF/NSs (69.76%). However, compared with pure SnO_2_ NF/NSs (30.24%), 1% GO@SnO_2_ NF/NSs showed larger O_ads_ percentage (50.86%). The chemisorbed oxygen species is crucial for the reaction with the target gas, which could significantly contribute to the improvement of gas-sensing performance [35].

To study the porous structure and surface area of pure SnO_2_ NF/NSs and 1% GO@SnO_2_ NF/NSs, the nitrogen adsorption–desorption measure was employed. As shown in Figure S1a, the corresponding N_2_ desorption–desorption isotherm curve of pure SnO_2_ NF/NSs and 1% GO@SnO_2_ NF/NSs showed a typical IV isotherm with a H3 hysteresis loop from 0.7 to 1 (P/P_0_), and the N_2_ adsorption quantity augments with the increase of relative pressure, which indicated the presence of the mesoporous structure in the as-prepared sensing materials. Detailed BET data are showed in Table S1. It is worth noting that 1% GO@SnO_2_ NF/NSs showed a BET surface area of 18.0 m^2^/g, which is larger than that of the pure SnO_2_ NF/NSs (16.1 m^2^/g). It demonstrated that BET surface area of the nanocomposites could further increase after the introduction of GO nanosheets. The larger BET surface area of 1% GO@SnO_2_ NF/NSs could provide more active sites for the adsorption of gas molecules, which could facilitate the improvement of gas-sensing performance [36,37]. With respect to the pore size distribution of these sensing materials, as can be observed in Figure S2b, the GO@SnO_2_ nanocomposites have a smaller pore size (25.7 nm) than that of pure SnO_2_ (37.8 nm), which may be ascribed to introduction and wrapping of GO on hierarchical SnO_2_. The larger BET surface area and smaller mesoporous channels can effectively promote diffusion and adsorption of gas molecules, improving gas-sensing performance of gas sensors.

### 2.2. Gas Sensing Properties

**Figure 5:**
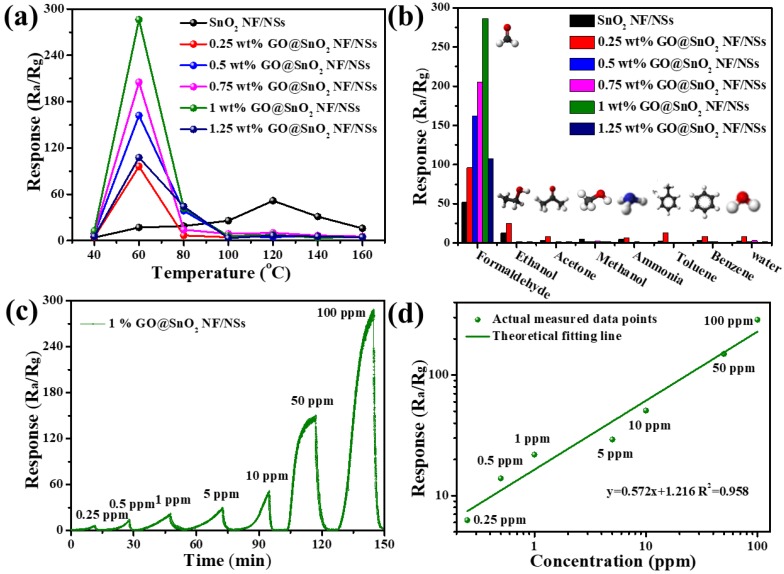
(**a**) The responses of gas sensors toward 100 ppm formaldehyde at different operation temperatures. (**b**) Responses of the gas sensors to different test gases at their respective optimal operating temperatures. (**c**) Response of 1% GO@SnO_2_ NF/NSs nanocomposites toward HCHO gas in concentration ranges of 0.25–100 ppm at 60 °C. (**d**) Linear approximation of the detection limit with 1% GO@SnO_2_ NF/NSs nanocomposite.

The working temperature has a great influence on gas response by controlling reaction kinetics of gas molecule and oxygen adsorbed on material surface [38]. To prove the influence of the operation temperature on the prepared sensing materials, the gas sensors based on the obtained sensing materials were prepared, and the gas-sensing performances of the pure SnO_2_ NF/NSs and GO@SnO_2_ NF/NSs with five different GO loading ratio (0.25%, 0.5%, 0.75%, 1% and 1.25% GO) toward 100 ppm HCHO were tested to confirm the optimal operation temperature. Many factors can influence the relationship between the adsorbed target gases and surface reactions [39,40]. At the beginning, HCHO molecules will react with the chemisorbed oxygen on the surface of sensing materials, and the redox reaction will be significantly activated at higher temperature. When the operation temperature is too high, the adsorption of HCHO molecules is likely to be suppressed, and the desorption may occur before the redox reactions, which will influence gas-sensing performance. Therefore, the optimal operation temperature is at an equilibrium point between adsorption and desorption processes. As shown in Figure 5a, the gas response values of gas sensors based on the pure SnO_2_ NF/NSs and GO@SnO_2_ NF/NSs nanocomposites toward 100 ppm HCHO gas at the operation temperature range of 40 to 150 °C were tested. The pure SnO_2_ NF/NSs sensor shows the highest response value of 51.76 at the operation temperature of 120 °C. However, compared with pure SnO_2_ NF/NSs based gas sensor, the GO@SnO_2_ nanocomposites based gas sensors show the highest response at a lower operation temperature of 60 °C, and the gas response values of 0.25%, 0.5%, 0.75%, 1%, 1.25% GO@SnO_2_ NF/NSs based gas sensors toward 100 ppm HCHO gas were 96.1, 162.1, 205.1, 286.2, and 107.7, respectively. The results can better prove the effect of GO in gas-sensing performance. It is obvious that GO@SnO_2_ NF/NSs nanocomposites with different GO doping amounts showed a lower optimal operation temperature (60 °C) than that of pure SnO_2_ NF/NSs (120 °C) owing to the introduction of GO, which lowered the activation energy in relation to the surface reaction. Hence, based on the above analysis, 1% GO was considered as the optimal doping amount in the GO@SnO_2_ NF/NSs nanocomposites at the optimal operation temperature of 60 °C. By taking Figure 5a into consideration, it has been demonstrated that appropriate GO loading amount in SnO_2_ NF/NSs not only enhanced the gas sensing performance to HCHO gas but also lowered the optimal operation temperature. The enhanced sensitivity could be ascribed to the loading of planar GO nanosheets, which constructed a 3D network and improved interconnectivity among hierarchical SnO_2_ NF/NSs [6]. However, excess GO loading amount may deteriorate the gas-sensing performance SnO_2_ NF/NSs to HCHO gas. The possible reason for it is that the excess GO nanosheets may cause a dramatic change of conductivity of sensing materials, which causes decrease of base resistance and degradation of gas-sensing performance of gas sensors [41]. Therefore, 1% GO@SnO_2_ NF/NSs was selected to further study other gas-sensing performance.

Selectivity is considered as a crucial part of gas-sensing performances. To further investigate the influence of GO in the selectivity of SnO_2_ NF/NSs, the gas responses of sensors based on 0.25% GO@SnO_2_ NF/NSs, 0.5% GO@SnO_2_ NF/NSs, 0.75% GO@SnO_2_ NF/NSs, 1% GO@SnO_2_ NF/NSs, 1.25% GO@SnO_2_ NF/NSs and the pure SnO_2_ NF/NSs toward 100 ppm of various gases were tested at different gas atmospheres, including ethanol (C_2_H_5_OH), acetone (CH_3_COCH_3_), methanol (CH_3_OH), ammonia (NH_3_), methylbenzene (C_7_H_8_), benzene (C_6_H_6_) and water (H_2_O). As exhibited in Figure 5b, these sensors showed a higher gas response for HCHO gas against other interfering gases. Especially, 1% GO@SnO_2_ NF/NSs nanocomposite sensor showed the highest response values of 286.2 to 100 ppm HCHO gas, which is nearly 50 to 100 times higher than other interfering gases, but it is only about 10 to 20 times for the pure SnO_2_ NF/NSs based sensors. Based on the analysis, the loading of GO in SnO_2_ NF/NSs can significantly enhance the selectivity of gas sensor to HCHO gas, and the 1% GO@SnO_2_ NF/NSs based gas sensor exhibited the best selectivity to HCHO gas in contrast with other GO@SnO_2_ NF/NSs nanocomposites and pure SnO_2_ NF/NSs. Many reasons can be argued to explain the enhancement of selectivity for GO@SnO_2_ NF/NSs nanocomposite-based sensors [42]. (i) The excellent selectivity of GO@SnO_2_ NF/NSs nanocomposites for HCHO gas detection could be ascribed to higher HCHO adsorption interaction between sensing materials. The sensing performance depends on many parameters between sensing materials and gas molecules, including absorption energy, distance of analysts above material surface, and charge transfer between the molecules and sensing materials. (ii) GO nanosheets generate more defects and functional groups, which acted as active sites for adsorption of gas molecules and could facilitate enhancement of the gas-sensing performance [43]. (iii) GO as a planar nanosheets can improve the degree of interconnection between SnO_2_ NF/NSs. Figure S3 exhibited the response/recovery curve of 1% GO@SnO_2_ NF/NSs toward 50 ppm HCHO gas. When the gas sensor based on 1% GO@SnO_2_ NF/NSs was tested in HCHO gas atmosphere of 50 ppm, the response and recovery times of gas sensor were 8.1 min and 3.0 min, respectively. Long response and recovery time can be ascribed to the low operation temperature that makes kinetic of the adsorption and desorption of oxygen and target gas on the surface of the lower sensing material, which causes a slow response and recovery process [44]. To investigate the relationship of HCHO gas concentration and response value of gas sensors, these gas sensors based on pure SnO_2_ NF/NSs and SnO_2_ NF/NSs with different GO loading amounts were tested in various HCHO gas concentrations. As shown in Figure S4, with increasing HCHO gas concentration from 0.25 to 100 ppm, the response value gradually increased. There are good relationships between HCHO gas concentration and response value of gas sensors. The dynamic responses of gas sensors based on pure SnO_2_ NF/NSs to 0.25–100 ppm HCHO gas at 60 °C were shown in Figure S5a. The gas sensor showed enhanced sensitivity as the HCHO concentration increased from 0.25 to 100 ppm. The response values of gas sensor were 2.1, 4.9, 6.7, 12.8, 17.0, 44.2, 60.8 and 157.5 for the sensors based on SnO_2_ NFs/NSs when tested in 0.25, 0.5, 1, 5, 10, 50 and 100 ppm HCHO gas. As shown in Figure S5b, with the increase of HCHO gas concentration, the sensor showed a linear relationship with R^2^ = 0.79 to HCHO gas concentration ranging from 0.25 to 100 ppm. Figure 5c showed dynamic responses of 1% GO@SnO_2_ NF/NSs based sensor 0.25 to 100 ppm HCHO gas at 60 °C. However, compared with pure SnO_2_ NF/NSs, 1% GO@SnO_2_ NF/NSs based sensor showed higher sensitivity to HCHO gas detection. The gas sensor showed enhanced sensitivity as the HCHO concentration increased from 0.25 to 100 ppm. The responses were 6.3, 13.9, 21.9, 29.2, 50.8, 149.7 and 287.6 for the sensors based on 1% GO@SnO_2_ NF/NSs when tested in 0.25, 0.5, 1, 5, 10, 50 and 100 ppm HCHO gas. To further analyze the relationship between the sensitivity and different concentrations of HCHO gas, the correlation lines between concentrations of HCHO gas and sensitivity of gas sensors based on 1% GO@SnO_2_ NF/NSs were fitted, as displayed in Figure 5d. Obviously, the gas response of gas sensor augment with the increase of HCHO gas concentrations in a linear relationship with R^2^ = 0.958 to HCHO gas concentration ranging from 0.25 to 100 ppm, and it showed an excellent linear relationship between HCHO gas concentration and sensitivity from low to high concentration of HCHO gas. It suggested that the response values gradually increased along with the augment of HCHO gas concentration, and the gas sensors based on the GO@SnO_2_ NF/NSs showed more significant improvement than the pristine SnO_2_ NF/NSs based sensor. Furthermore, the gas sensors based on these gas sensing materials revealed an excellent linear detection ranging from 0.25 ppm to 100 ppm.

The gas-sensing performance of SnO_2_ NF/NSs can be improved by introduction of GO nanosheets, so many studies based on GO and SnO_2_ have been reported. The recently reported HCHO gas sensors based on GO and SnO_2_ have been summarized. As can be seen in Table 2, the GO@SnO_2_ NF/NSs based sensor showed better gas-sensing performance, which has a lower operation temperature and a higher gas response. The gas sensors based on GO@SnO_2_ NF/NSs nanocomposites show better sensing performance, which provides an effective and facile method for the development of HCHO gas sensor with high response and excellent selectivity at a lower operation temperature.

### 2.3. Gas Sensing Mechanism

**Figure 6:**
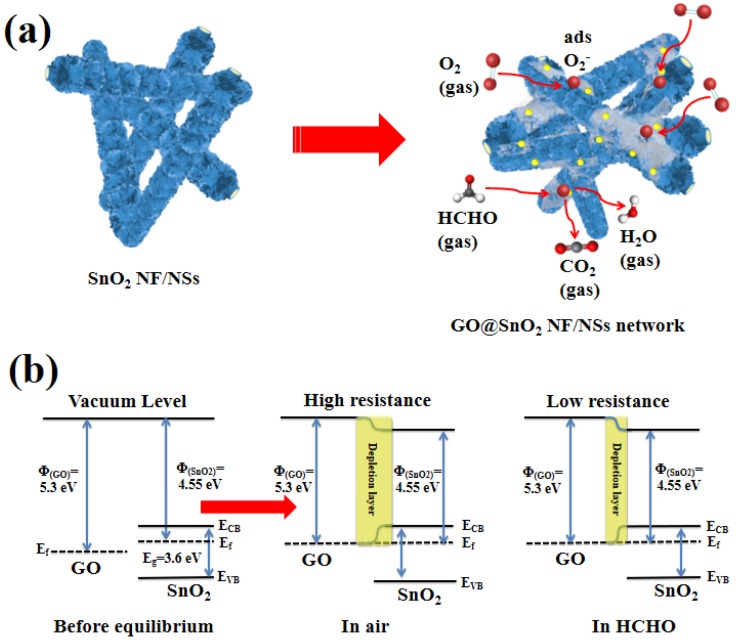
Schematic illustration of HCHO gas sensing mechanism for (**a**) pure SnO_2_ NF/NSs and GO@SnO_2_ NF/NSs nanocomposite; (**b**) the energy band structures of GO@SnO_2_ NF/NSs nanocomposite in different gas atmospheres.

The sensing process of the nanocomposites is mainly based on the chemical reaction between the sensing materials and different gas molecules. As shown in Figure 6a, when the gas sensors were exposed to air atmosphere, oxide molecules chemisorbed on the active surface of sensing materials were changed into various oxide species (O_2_^−^, O^−^, or O^2−^) [52]. The chemisorbed oxygen species depended on their working temperature. The continual transport of electrons from conduction band of the sensing materials to the as-chemisorbed oxide molecules leads to augment of electron depletion layer on the surface of sensing materials and increase of resistance. The relative reaction process between the sensing materials and oxide molecules can be represented as the following Equations (1) to (4).

O_2_ (gas) → O_2_ (ads) (1)

O_2_ (ads) + e^−^ → O_2_^−^ (ads)     (Top < 100 °C) (2)

O_2_^−^ (ads) + e^−^ → 2O^−^ (ads)     (100 °C < Top < 300 °C) (3)

O^−^ (ads) + e^−^ → O^2−^ (ads)     (300 °C < Top) (4)

Based on the analysis in Figure 5a, the gas-sensing performance of these gas sensors was studied under the operation temperature of 60 °C. Therefore, the oxide molecules chemisorbed on the surface of sensing materials were ionized into O_2_^−^. When the HCHO gas was introduced, the HCHO gases would react with the chemisorbed oxide ions on the surface of sensing materials, and the trapped electrons would be released back to the conduction band of sensing materials. The relative reaction process can be represented as the following Equation (5) [7,53,54].

HCHO (ads) + 2O^−^ (ads) → CO_2_ (gas) + H_2_O (gas) + 2e^−^ (5)

Compared with the pure hierarchical SnO_2_ NF/NSs, the gas sensors based on GO@SnO_2_ NF/NSs nanocomposites exhibited better gas-sensing performance. The enhanced sensing performance is attributed to the following reasons.

(i) Enhanced specific surface area played a critical role in the improvement of gas-sensing performance toward HCHO gas. As discussed about the BET surface area in Figure S2 and Table S1, GO@SnO_2_ NF/NSs produced a larger specific surface area (18.0 m^2^/g) than that of pure SnO_2_ NF/NSs (16.1 m^2^/g), which indicated that more active adsorption sites existed on the surface of GO@SnO_2_ NF/NSs. The improved surface activity can make more oxide molecules adsorbed and ionized on the surface of sensing materials. Therefore, larger specific surface area of GO@SnO_2_ NF/NSs is all beneficial to facilitate the improvement of the sensing properties for HCHO gas [52].

(ii) The introduction of GO can act as an active site for the absorption of gas molecules and influence the transfer of electrons between the GO and SnO_2_. The existence of GO can provide more active sites for the reaction with target gas molecules. Strong gas adsorption capacity of GO nanosheets can effectively facilitate the chemical reaction, which can improve the sensing performance of gas sensors. Moreover, due to the different work functions of GO and SnO_2_, when the GO and SnO_2_ are in contact, the electrons will be transferred from the SnO_2_ with lower work functions of 4.55 eV to the GO with higher work functions of 5.3 eV until an equalization of the Fermi levels, which causes the band bend of p-n heterojunction between the GO and SnO_2_ [55]. Schematic illustration of HCHO gas sensing mechanism was shown in Figure 6b. Transfer of a large number of electrons results in the increase of electron depletion layer in SnO_2_ to some extent, which could lead to a higher initial resistance. Therefore, when the gas sensors are exposed to HCHO gas atmosphere, it will cause huge resistance changes [6,41,56].

(iii) The potential barrier at the interfaces between hierarchical SnO_2_ NF/NSs and GO was modified because abundant oxygen-containing functional groups exist in GO, including carboxylic, hydroxyl, and epoxy groups, and some chemical interactions between GO and hierarchical SnO_2_ NF/NSs would increase the electrical conductibility of GO and influence the carrier concentration of hierarchical SnO_2_ NF/NSs [57,58].

## 3. Materials and Methods

### 3.1. Materials

Poly (vinyl pyrrolidone) (PVP, Mw = 1,300,000), *N,N*-dimethylformamide (DMF) and ethanol (EtOH) (99.0%) were purchased from Shang Hai Aladdin Industrial Co. Ltd., China. Stannous chloride (SnCl_2_·2H_2_O) and sodium citrate (Na_3_C_6_H_5_O_7_·2H_2_O) were obtained from Sinopharm Chemical Reagent Co. Ltd., China. The above chemical reagents were analytical grade and used without further purification.

### 3.2. Preparation of Sensing Materials

**Scheme 1:**
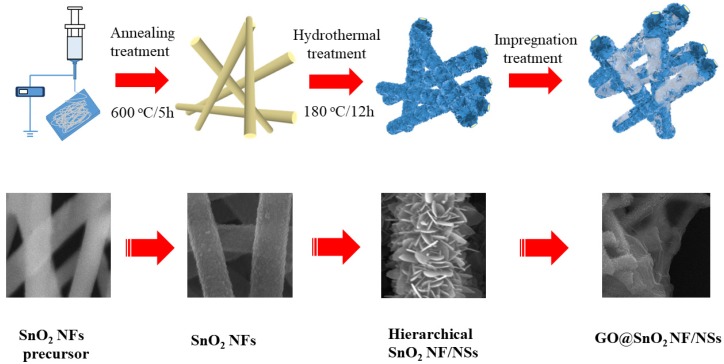
A schematic demonstration of the preparation process of GO@SnO_2_ NF/NSs nanocomposites.

GO@SnO_2_ NF/NSs nanocomposites were synthesized via electrospinning and further hydrothermal method. In a typical synthesis, Scheme 1 showed the schematic diagram of the preparation of SnO_2_ nanofiber/nanosheets materials. SnO_2_ NF/NSs was prepared according to our previous work [57].

The preparation of GO@SnO_2_ NF/NSs nanocomposites: GO was prepared from graphite powder by Hummers’ method [6]. GO (12 mg) was added into 40 mL ethanol under ultrasound for 1 h to obtain uniform solution. Under ultrasonic, a certain amount of the as-prepared GO solution was dropwise added into SnO_2_ NF/NSs under stirring with grass rob. The GO@SnO_2_ NF/NSs nanocomposites were obtained by drying at 60 °C for 12 h. The nanocomposites with different loading amounts of GO to the obtained sensing material (0.25%, 0.5%, 0.75%, 1% and 1.25%) were prepared, respectively.

### 3.3. Characterization

To analyze the microstructures and morphologies of the prepared products, FE-SEM (FEI, Quanta FEG 450, Hillsboro, OR, USA) and Tecnai G220S-Twin transmission electron microscope were used at an accelerating voltage of 30 and 120 kV. XRD (Bruker, D8 Advance, Karlsruhe, Germany) with Cu-Kα (λ = 0.15418 nm) radiation in the range of 10° to 70° at room temperature was conducted to analyze the crystalline structures of the prepared products. X-ray photoelectron spectroscopy (XPS, Thermo Scientific, ESCALAB 250, Waltham, UK) with Mg Kα radiation was used to analyze elemental compositions and chemical states of the prepared products. QUADRASORB SI gas adsorption analyzer (N_2_ as adsorbate and operation temperature: −196 °C) was used to measure the specific surface area and porosity of the prepared products. Raman Microscopy (Horiba, LabRAM HR Evolution, Villeneuve-d’Ascq, France) with an excitation wavelength of 633 nm was conducted to analyze the Raman spectra.

### 3.4. Gas Sensor Fabrication and Measurement

**Figure 7:**
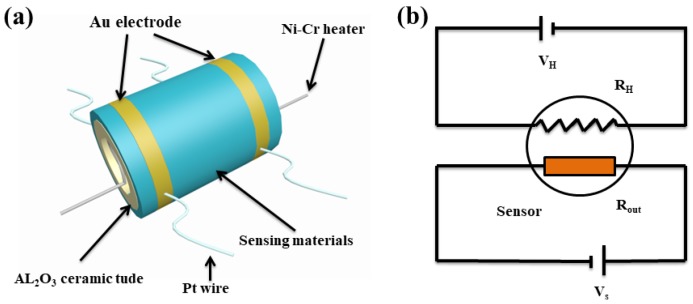
(**a**) The schematic structure and (**b**) the working principle of the gas-sensing test system of the gas sensor.

A paste was obtained by mixing the prepared GO@SnO_2_ NF/NSs with a certain amount of water at a ratio of 4:1, and a ceramic tube with a pair of gold electrodes was coated by the prepared sensing material with a small brush. A Ni-Cr heating inserted into the ceramic tube was applied to provide a certain operation temperature by a temperature controller. The general structure of gas sensor and the test circuit were displayed in Figure 7a,b, respectively. For the reducing gases and n-type metal oxide semiconductor, the response of gas sensors was defined as the ratio of R_a_ and R_g_, where R_a_ and R_g_ are the resistance in air and target gas, respectively. The times taken by the resistance ranging from R_a_ to R_a_ − 90% (R_a_ − R_g_) and from R_g_ to R_g_ + 90% (R_a_ − R_g_) are defined as the response and recovery times when the sensor is exposed to the target gas and retrieved from the target gas, respectively.

## 4. Conclusions

In summary, GO@SnO_2_ NF/NSs nanocomposites have been successfully prepared. The microstructures, morphologies, compositions and surface areas of the obtained materials were investigated by using a series of characterization methods. The obtained 3D hierarchical GO@SnO_2_ NF/NSs nanocomposites possess larger specific surface areas and rich SnO_2_-GO interfaces. Compared with pristine SnO_2_ NF/NSs, GO@SnO_2_ NF/NSs nanocomposites showed higher response value and better selectivity for HCHO, and the optimum loading ratio of GO in the GO@SnO_2_ NF/NSs nanocomposites is 1 wt %. Especially, 1% GO@SnO_2_ NF/NSs nanocomposite showed the highest response value for HCHO (R_a_/R_g_ = 280 to 100 ppm) at a lower operation temperature of 60 °C. The gas sensors for HCHO show a low detection limit of 0.25 ppm. The enhanced gas-sensing performances are mainly ascribed to larger specific surface area, electric regulation effects of GO with rich functional groups and the synergistic effects of hierarchical SnO_2_ NF/NSs and GO. These GO@SnO_2_ nanocomposites are promising for high-performance gas sensors applied in various fields such as environmental protection.

## Figures and Tables

**Table 1:** The XPS spectra results of pristine SnO_2_ NF/NSs and 1% GO/SnO_2_ NF/NSs.

Sensing Materials	Sn 3d	Binding Energy (eV)	Oxygen Species	Relative Percentage (%)	Binding Energy (eV)
SnO_2_ NF/NSs	Sn^4+^ 3d_3/2_	494.7	O_lat_	69.76	530.2
	Sn^4+^ 3d_5/2_	486.3	O_ads_	30.24	531.3
1% GO@SnO_2_ NF/NSs	Sn^4+^ 3d_3/2_	494.9	O_lat_	49.14	530.3
	Sn^4+^ 3d_5/2_	494.5	O_ads_	50.86	531.3

**Table 2:** Sensing performance of recently reported SMO-based gas sensors to HCHO gas.

Sensing Materials	T (°C)	LOD (ppm)	Concentration (ppm)	Response (R_a_/R_g_)	References
GO/SnO_2_	120	0.25	100	32	[6]
Flower-like SnO_2_	240	1	100	24.8	[45]
GO-SnO_2_	150	10	200	90	[46]
Porous PtO_2_/SnO_2_	100	0.1	100	70	[47]
SnO_2_@graphene	120	1	100	45	[48]
Graphene/ZnO	200	2	100	12	[49]
In_2_O_3_/SnO_2_	275	0.5	10	8.7	[50]
In_2_O_3_@rGO	225	5	100	1.8	[51]
VG/SnO_2_	160	0.02	100	138	[5]
GO@SnO_2_ NF/NSs	60	0.25	100	280	This work

## Supplementary Materials

The following are available online, Figure S1. Sn 3d of SnO_2_ NF/NSs and 1% GO@SnO_2_ NF/NSs nanocomposite, Figure S2. BET surface area characterization. (a) Nitrogen (N2) adsorption-desorption isotherms, and (b) corresponding pore size distribution curves of SnO2 NF/NSs and 1% GO@SnO2 NF/NSs nanocomposites, Figure S3. The gas response of 1% GO@SnO2 NF/NSs nanocomposites toward 50 ppm formaldehyde gas at the optimal operation temperature of 60 °C, Figure S4. The relationship of these gas sensors toward different HCHO gas concentration (0.25–100 ppm) at 60 °C, Figure S5. (a) response of pure SnO_2_ NF/NSs toward HCHO gas in concentration ranges of 0.25–100 ppm at 60 °C, (b) the relationship of these gas sensors based on pure SnO_2_ NF/NSs toward different HCHO gas concentration (0.25–100 ppm) at 60 °C, Table S1. structural character of the prepared pure and nanocomposite sensing materials.
