# Supplementary material for: Graphene Oxide@3D Hierarchical SnO2 Nanofiber/Nanosheets Nanocomposites for Highly Sensitive and Low-Temperature Formaldehyde Detection
Source: Molecules. 2019 Dec 20;25(1):35. doi: 10.3390/molecules25010035 (PMC6983051; doi:10.3390/molecules25010035)
Supplement: Supplementary file 1 [file molecules-25-00035-s001.pdf]

Supplementary Material

Graphene oxide@3D hierarchical SnO_2 nanofiber/nanosheets nanocomposites for highly sensitive and low-temperature formaldehyde detection

Kechuang Wan, Jialin yang, Ding Wang* and Xianying Wang

S1. The test conditions of gas sensors

The RH% of test environment was monitored by a humidity sensor within the CGS-8 gas sensing measurement system. These gas sensors were tested under relative humidity (RH%) of 40-50 %. The gas sensing performance of gas sensor was measured by a static test system (Elite tech co. LTD) and the test gases were obtained by static distribution method. The different concentrations of vapors were obtained by injecting liquid or gas of volume Q into a testing chamber. The volume Q can be determined by

$$Q = \frac{V \times C \times M}{22.4 \times d \times \rho} \times 10^{-9} \times \frac{273 + T_R}{273 + T_B} \quad (1)$$

Here, V, C, M, d, ρ, TR, and TB are the test chamber volume, vapor concentration (ppm), molecular mass, liquid density, liquid purity, environmental temperature, and temperature in the testing chamber, respectively. HCHO vapor was obtained by evaporating 40 % HCHO solution. Therefore, the gas sensitivity of gas sensors was obtained in regular laboratory air.

S2. Sn 3d of SnO_2 NF/NSs and 1% GO@SnO_2 NF/NSs nanocomposite

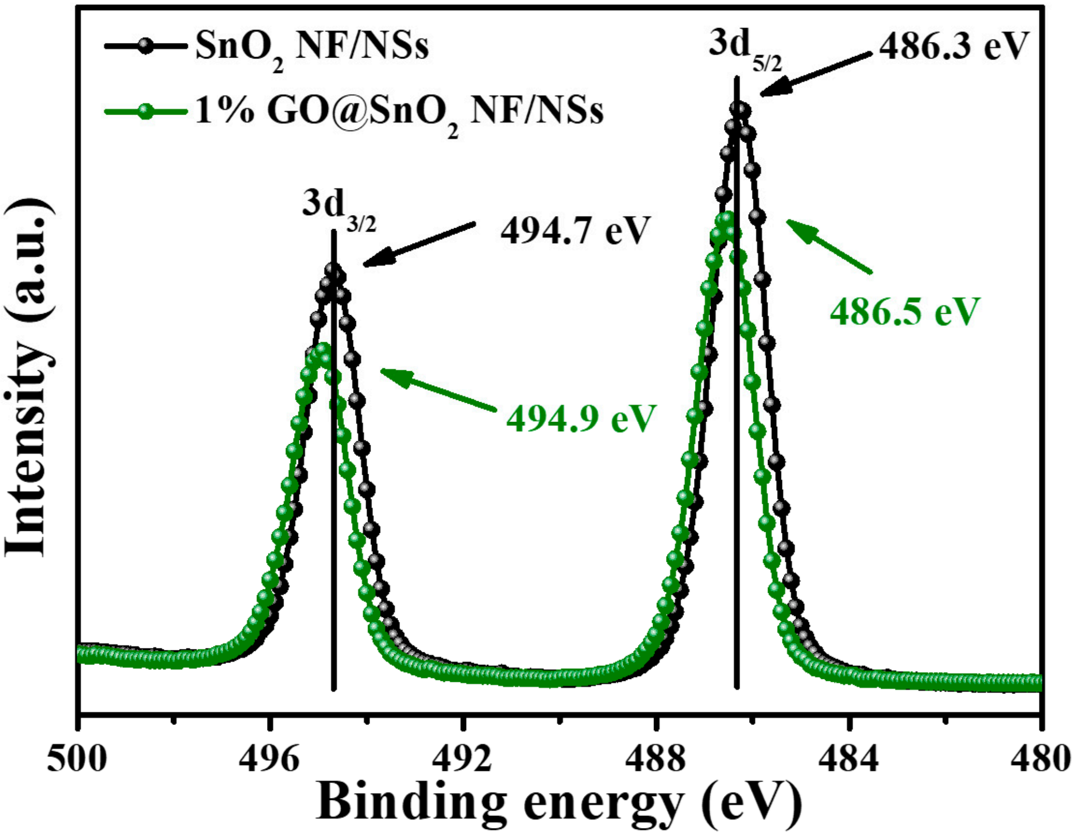

Figure 1. Sn 3d of SnO_2 NF/NSs and 1% GO@SnO_2 NF/NSs nanocomposite.

S3. The structure characterization of materials

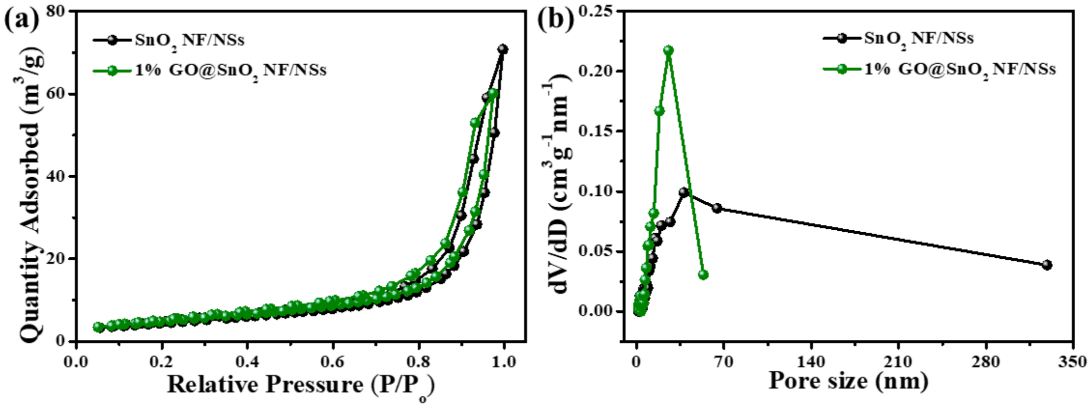

Figure 2. BET surface area characterization. (a) Nitrogen (N_2) adsorption-desorption isotherms, and (b) corresponding pore size distribution curves of SnO_2 NF/NSs and 1% GO@SnO_2 NF/NSs nanocomposites.

S4. The sensing performance of gas sensor

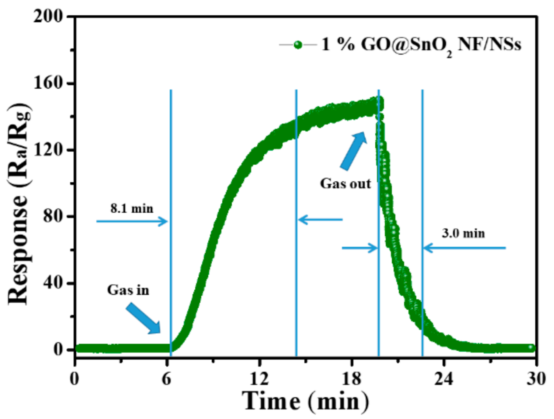

Figure 3. The gas response of 1% GO@SnO_2 NF/NSs nanocomposites toward 50 ppm formaldehyde gas at the optimal operation temperature of 60 °C.

S5. The relationship of these gas sensors toward different HCHO gas concentration

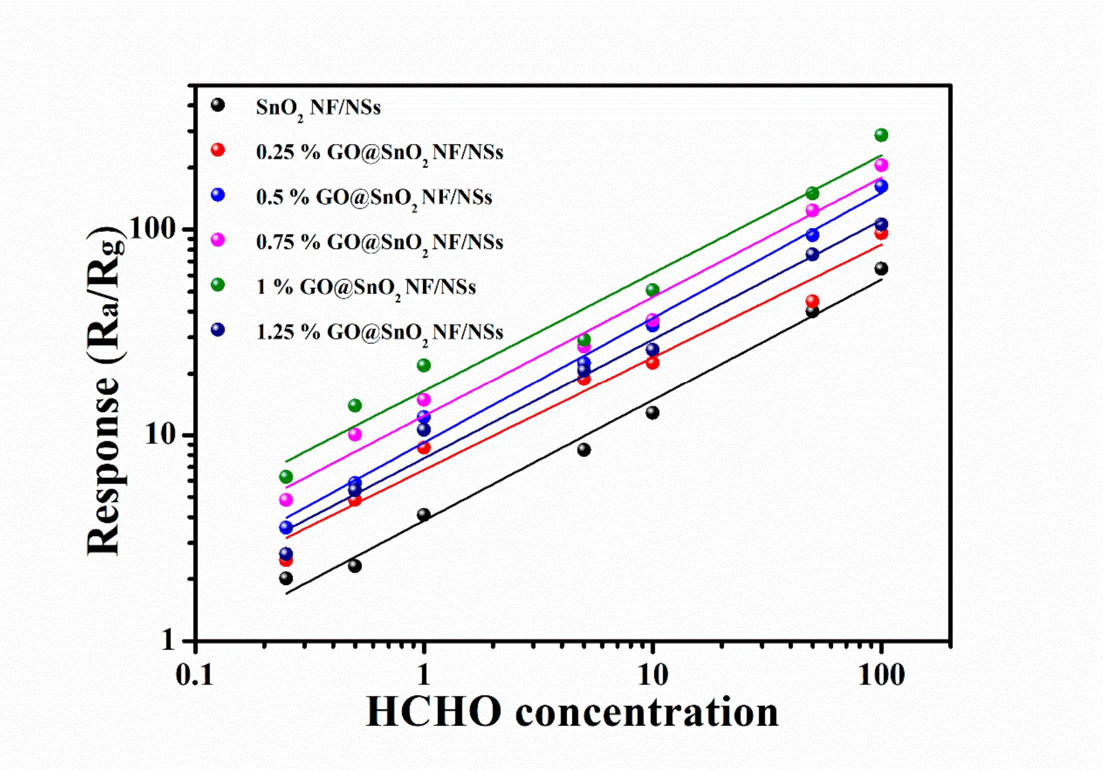

Figure 4. The relationship of these gas sensors toward different HCHO gas concentration (0.25 -100 ppm) at 60 °C.

S6. Responses of pure SnO_2 NF/NSs toward different HCHO gas concentration

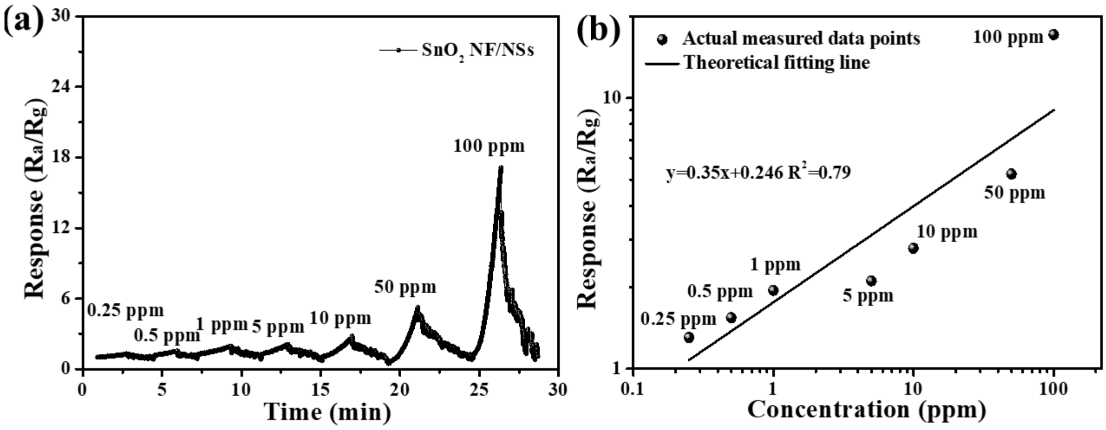

Figure 5. (a) response of pure SnO_2 NF/NSs toward HCHO gas in concentration ranges of 0.25 -100 ppm at 60 ℃, (b) the relationship of these gas sensors based on pure SnO_2 NF/NSs toward different HCHO gas concentration (0.25 -100 ppm) at 60 °C.

Table 1. structural character of the prepared pure and nanocomposite sensing materials.

Sensing materials	Crystallite size (nm)	Surface area (m^2g^{-1})	Pore size (nm)	Pore volume (cm^3g^{-1})
SnO_2 NF/NSs	14.1	16.1	25.7	0.087
1% GO@SnO_2 NF/NSs	16.0	18.0	37.8	0.076
